# Contextual Effects of Scene on the Visual Perception of Object Orientation in Depth

**DOI:** 10.1371/journal.pone.0084371

**Published:** 2013-12-31

**Authors:** Ryosuke Niimi, Katsumi Watanabe

**Affiliations:** 1 Graduate School of Humanities and Sociology, The University of Tokyo, Tokyo, Japan; 2 Research Center for Advanced Science and Technology, The University of Tokyo, Tokyo, Japan; University of Montreal, Canada

## Abstract

We investigated the effect of background scene on the human visual perception of depth orientation (i.e., azimuth angle) of three-dimensional common objects. Participants evaluated the depth orientation of objects. The objects were surrounded by scenes with an apparent axis of the global reference frame, such as a sidewalk scene. When a scene axis was slightly misaligned with the gaze line, object orientation perception was biased, as if the gaze line had been assimilated into the scene axis (Experiment 1). When the scene axis was slightly misaligned with the object, evaluated object orientation was biased, as if it had been assimilated into the scene axis (Experiment 2). This assimilation may be due to confusion between the orientation of the scene and object axes (Experiment 3). Thus, the global reference frame may influence object orientation perception when its orientation is similar to that of the gaze-line or object.

## Introduction

Human vision obtains a variety of information from objects. For example, object orientation is essential for spatial perception, object-directed behaviors (e.g., tool use), and comprehension of environment containing multiple objects. Numerous studies have discussed the mechanisms of object recognition/identification. However, how the visual system determines object orientation in three-dimensional (3D) space is less understood.

How accurately does the visual system perceive object orientation? In principle, 3D orientation estimation from a 2D retinal image is an ill-posed problem (e.g., [1]). Therefore, it is not surprising that perceived object depth orientation is imprecise. Several studies have demonstrated that the perception of oblique object orientation (e.g., three-quarter view) is inaccurate. First, visual sensitivity to object orientation differences is lower for oblique orientations than for cardinal orientations (front, profile) for everyday objects [2] and human heads [3]. This is akin to the oblique effect in the perception of line orientation on the front parallel plane [4], [5]. Second, perception of object orientation deviates systematically from physical orientation. In Niimi and Yokosawa's study [6], participants observed object images presented on a computer screen and estimated the objects' orientation in depth (i.e., azimuth angle). Their results showed that oblique orientations yielded significant perceptual biases toward the profile view. For example, an object oriented at 27° (zero indicates the front) was estimated to be oriented at 39.7° on average (i.e., the bias was 12.7°), suggesting that the rotation angle from the front view was overestimated. Similar biases have also been reported for the slant estimation of simple 3D objects [7]–[9]. One possible explanation for the bias in oblique orientation estimation is the low visual similarity between frontal views and three-quarter views, as it is well known that the frontal orientation often yields accidental and unfamiliar views [10], [11].

Orientation judgment requires a processing reference frame, usually the egocentric reference frame. Previous studies have tested the perception of object orientation based on the egocentric reference frame (i.e., determining object orientation relative to the gaze line) and presented object stimuli on a blank background. However, in daily visual experiences we observe objects embedded in visual scenes, which contain rich spatial information—including global (or allocentric) reference frames. It is known that a scenic background is automatically processed during visual object perception [12], [13].

Global reference frames may influence the perception of object orientation in two ways. First, global reference frames provide rich spatial information and may improve depth perception. If biases in oblique orientation perception when objects are presented on blank backgrounds are partly due to a lack of global reference frames, then object orientation perception may be more precise when an appropriate global reference frame is provided. Alternatively, global reference frames may induce a contextual effect that biases or distorts object orientation perception.

Many studies have demonstrated that contextual stimuli such as background scene play the role of a global reference frame, and bias human performance on spatial tasks related to orientation. For example, surrounding stimuli alter orientation judgments of 2D shapes [14], [15]. Perception of slant defined by binocular disparity is affected by flanking contextual surfaces [16]. Visual backgrounds (e.g., picture of a room) tilted in the front parallel plane bias observers' judgments of subjective upright [17]–[19]. Memories of spatial layout are organized in terms of global reference frames [20]–[22]. Moreover, it was shown that task performance related to perception of depth orientation was biased when the room orientation was not aligned with participants' gaze line [23], [24]. Although the experimental tasks in these studies (parallelity judgments or pointing) did not measure perceived object orientation directly, the results led us to hypothesize that a global reference frame may influence the perception of object orientation in 3D space.

The current study examined the effect of a background scene that suggests a global reference frame (e.g., room, street) on perceived object depth orientation. Although the actual environment does not always provide such salient reference frame (e.g., desert, deep forest), we focused on simplified situations that have salient reference frames. We asked participants to evaluate object orientation while manipulating the background images. The first goal was to contrast orientation judgments of objects presented with a scene and those presented without a scene (i.e., shown on a blank background). For example, the presence of an apparent global reference frame that is aligned with participants' gaze lines might improve depth perception and thus reduce bias in judgments of oblique orientations. Second, we varied the axis orientation of the scene and examined whether an oblique scene axis would produce a contextual effect, and thus bias perception of object orientation.

## Experiment 1

In Experiment 1, participants viewed objects presented on a computer screen and evaluated their depth orientation (i.e., azimuth angle). We measured estimated object orientations and their deviations from the true object orientations when (1) the scene was absent (blank background), (2) the scene was present and its orientation was aligned with the gaze line, and (3) the scene was present but misaligned with the gaze line. We used scene stimuli with dominant structures (street, building, wall) that regulated orientation of other objects in the scene. The dominant structures provided the principal axis as a global reference frame, and then, we defined scene orientation as the orientation of the axis.

### Method

#### Participants

Nineteen individuals (13 female, 6 male; mean age 23.4 years; range 19–44 years) were paid for their participation in this experiment. They all reported normal or corrected-to-normal visual acuity.

#### Stimuli

Stimuli were colored images generated by 3D graphic software (Shade 9, e-frontier Inc., Tokyo). Cast shadows were not rendered. We adopted a 3D model data of 24 common objects (18 for experimental trials and 6 for practice trials), which have been used in previous studies [2], [6]. All the objects had clear frontal orientations and upright positions. Objects with a thin or elongated shape (e.g., dish, stapler) were avoided. We included six wide objects (e.g., bench), six high objects (e.g., standing fan), and six deep objects (e.g., tricycle). See Figure S1 for the entire list.

**Figure 1:**
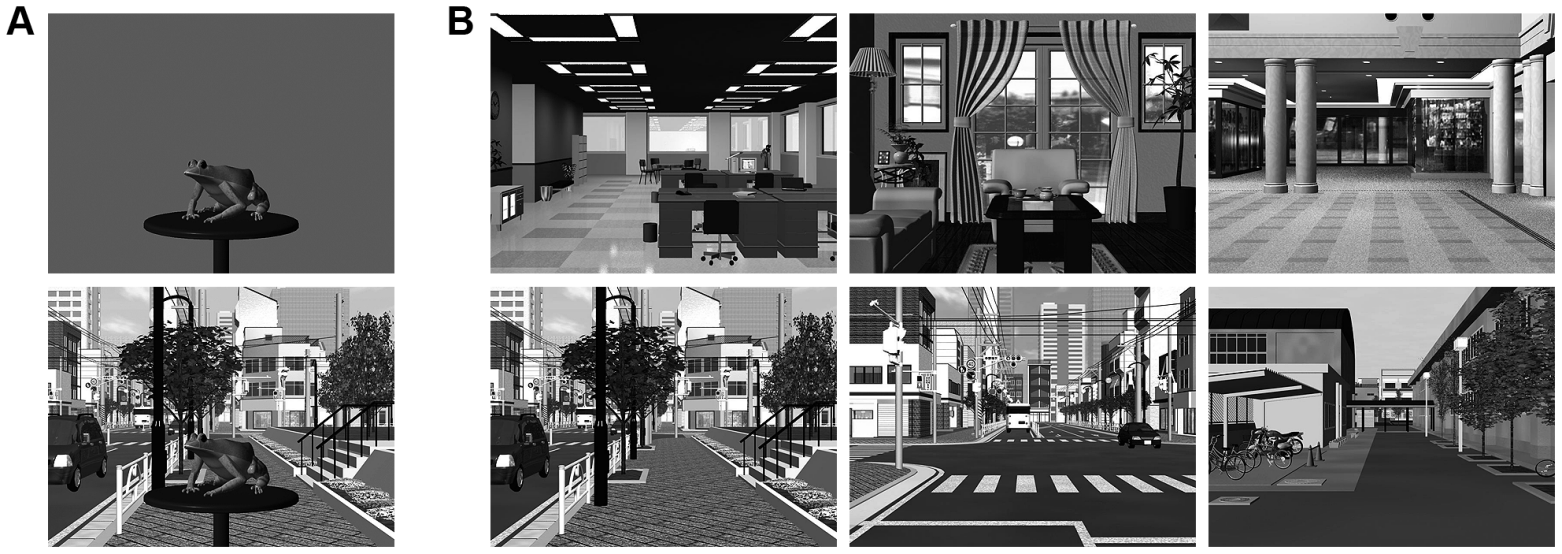
Examples of the stimuli. A. Scene Absent condition and Scene 0° condition in Experiment 1. B. Six scenes shown in gaze-aligned orientations. All stimulus images were presented in color during the experiment.

We prepared six scenes (three indoor and three outdoor) that had obvious global reference frame axes (see Figure 1B). These scenes were constructed by assembling 3D models of objects available in commercial datasets. We put a round table in front of the viewpoint and placed the target object on the table (Figure 1A); the objects and the scene were rendered into stimulus images. The position of the table relative to the viewpoint was fixed. As seen in Figure 1, the scene's depth is defined predominantly by the perspective and the perspective does not represent the principal characteristic of the depth for the object's orientation. We adopted various objects and scenes in order to reduce any object/scene-specific effect. However, the semantic consistency of object and scene was not controlled (e.g., a sea turtle with a street scene is inconsistent but allowed in this experiment).

The objects and scenes were rotated about the vertical axis to manipulate depth orientation. The axis of rotation was along the center of the table.

#### Apparatus

Participants observed the stimulus images presented on a computer screen (22-in CRT), binocularly. No stereoscopic device was used. Consequently, we studied the effect of pictorial depth information on the perception of object orientation in the same manner as in our previous studies [2], [6]. However, it should be noted that a binocular disparity might reduce bias in 3D orientation perception [25].

If an observer views a perspective picture from a viewpoint that deviates from the viewpoint from which the picture was taken, the perceived 3D space may be distorted [9], [26]–[29]. To avoid this problem, we matched the participants' perspective and the perspective of our “virtual camera” in the 3D graphic software used to render the stimulus images. The participants' gaze line was roughly directed to the center of the screen. The stimulus images subtended 36.2° in width and 27.6° in height (the viewing distance was 57 cm). This field of view was the replication of the virtual camera. The horizontal field of view was roughly matched to that of a 55-mm focal length for a 35-mm film.

**Figure 2:**
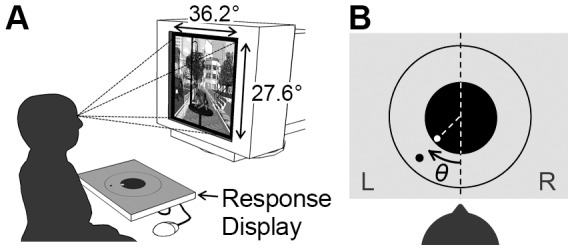
Schematic illustration of the apparatus used in Experiments 1 and 2. A. Participants rotated the disk on the horizontal response display so that the disk orientation matched the perceived depth orientation of the object. B. On the response display, a white dot indicated the frontal orientation of the response disk. The disk orientation *θ* was measured as evaluated orientation. The characters, arrow, and dotted lines did not appear in the experiments.

Another screen, the response display, was located horizontally in front of the participants (Figure 2). Participants were asked to adjust the orientation of a dark disk on the response display (response disk) so that it matched the orientation of the object. A white dot on the edge of the response disk marked the front of the disk. A mouse cursor was displayed as a black dot, and participants used the mouse to rotate the response disk by clicking and dragging. No participant reported difficulty in using the response display after completing practice trials.

#### Design and Procedure

The task was identical to that of a previous study [6]; participants evaluated the object orientation by adjusting the orientation of the response disk. They were instructed to respond as accurately as possible, and were allowed to adjust the disk orientation as many times as they liked. They proceeded to the next trial by clicking the peripheral area (i.e., area outside the large circle) in the response display. The response disk orientation (*θ* in Figure 2B) was recorded as the evaluated orientation of the trial. No numerical expression regarding the orientation (e.g., “frontal orientation is zero degrees”) was used in the experiment. Participants were asked to “imagine the top view of the object and match the disk orientation with the object orientation, so that the white dot of the disk indicates the object's heading.”

We tested object orientations of 9°, 27°, and 45° (0° =  front), since a previous study showed that the magnitude of the perceptual bias was maximal for object orientations of around 30°. Participants were not informed of these orientations; they were told, “the objects will be shown in various orientations.” Rotations were made in either a leftward or a rightward direction. Figure 1A shows an example of an object rotated 45° to the left.

In the Scene Absent condition, an object and the table were presented on a uniform gray background (see Figure 1A). In the other conditions, background scenes were present and their orientations varied (0°, +/−9°, and +/−18°). Note that Scene 0° indicates that the scene axis was aligned with the participants' gaze line. Positive orientations indicated that the scene was rotated in the same direction as the object, while negative orientations indicated that the scene was rotated in the opposite direction of the object. We averaged the data from conditions in which the spatial layout of the orientations was mirror-symmetric; for example, both [object  = 9° right, scene  = 18° left] and [object  = 9° left, scene  = 18° right] were considered as a single condition and denoted as [object  = 9°, scene  = −18°].

The object orientations (9°, 27°, 45°) and the scene conditions (Absent, 0°, +/−9°, +/−18°) yielded 18 trial types that were repeated for both left object orientations and right object orientations, resulting in 36 trial types. Each participant performed 648 trials (36 trial types ×18 objects). The six scenes were randomly assigned to the 36 trial types. Trial order was randomized. Prior to the experimental trials, participants completed 12 practice trials. The experiment lasted approximately 80 minutes.

#### Ethical Statements

This experiment, as well as the subsequent experiments, were approved by the institutional review board (IRB) of Research Center for Advanced Science and Technology, The University of Tokyo, and conducted in accordance with the Code of Ethics and Conduct (2009) of the Japanese Psychological Association. Written informed consent was obtained from all participants in advance.

### Results

We did not find any clear difference in scene effect by object shape (wide/high/deep) and scene category (indoor/outdoor). Therefore, we averaged the data over objects and scenes in the following analyses.

**Figure 3:**
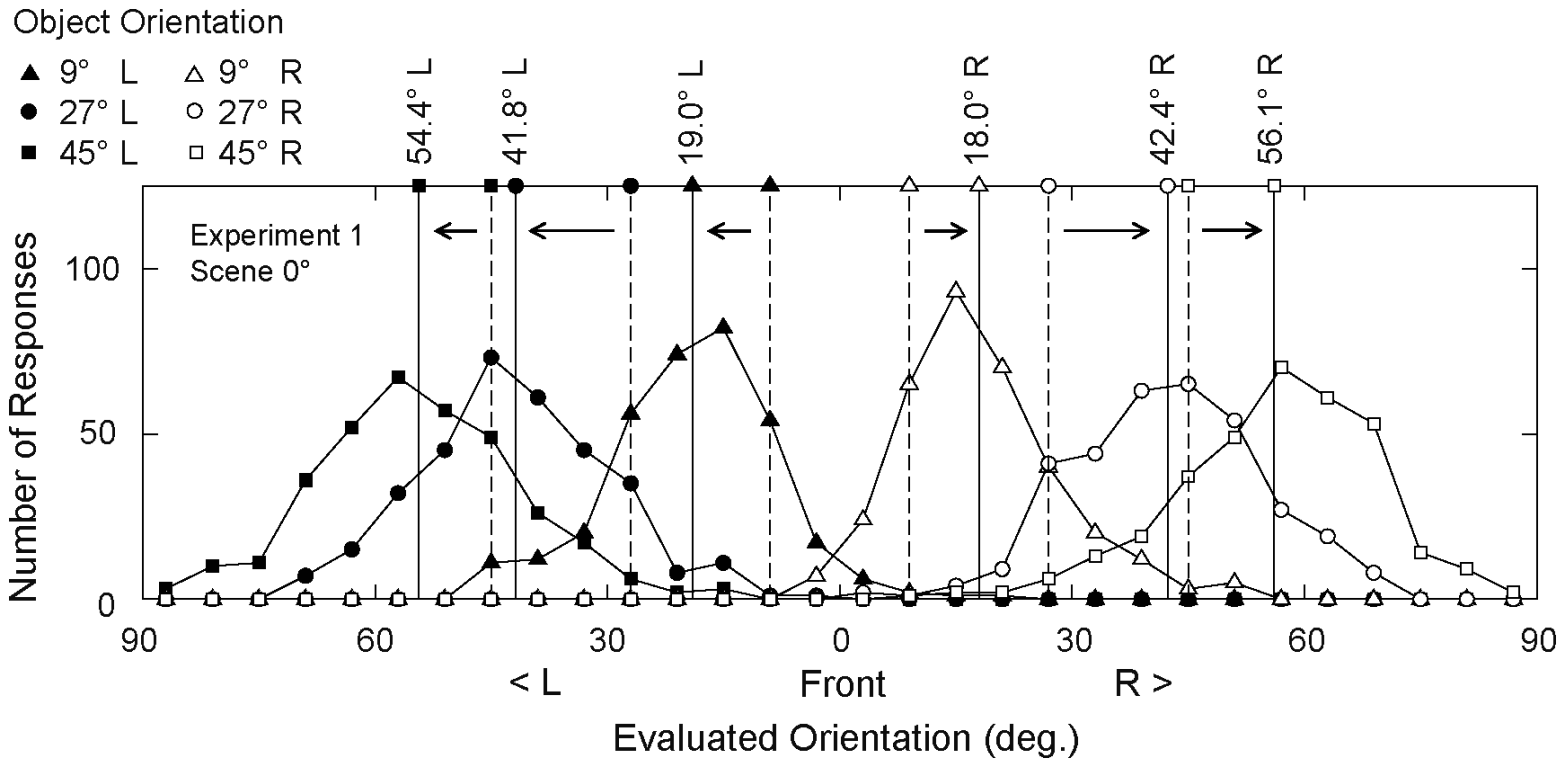
Frequency distributions of evaluated orientation in Scene 0° condition, Experiment 1. Bin width is 6°. The six distributions correspond to the six object orientation conditions, as indicated by the symbols. The vertical dotted lines mark the positions of stimulus object orientation (the “true” orientation). The vertical solid lines mark the mean evaluated orientations. The evaluated orientations were biased toward profile, as indicated by the arrows. Since the results were symmetric, we ignored left/right difference and symmetric object orientation conditions were merged into single condition in the following analyses. L, left; R, right.

First, we confirmed whether the bias toward profile that Niimi and Yokosawa (2009) reported would be replicated in the current experiment. Figure 3 shows the distributions of evaluated orientation in the Scene 0° condition. On average, the evaluated orientations systematically deviated from the “true” orientations of the stimulus objects. For instance, the mean evaluated orientation for the 9° left condition was 19.0° left, namely, a 10.0° bias toward profile. In all the object orientation conditions, the bias was toward profile. This pattern of bias was consistent with our previous study [6], in which the background scene was absent. As evident in Figure 3, the results were clearly symmetric. Therefore, we ignored the left/right difference and considered each pair of symmetric conditions (e.g., 9° left and 9° right) as a single condition in the following analyses.

We measured “bias” by subtracting the true object orientation from the evaluated orientation. Note that a positive value of bias indicates a bias toward profile, and a negative value of bias indicates a bias toward front. When the absolute value of the bias was larger than 45°, we regarded the response as an error and excluded it from further analysis. The mean error rate was 1.3%.

**Figure 4:**
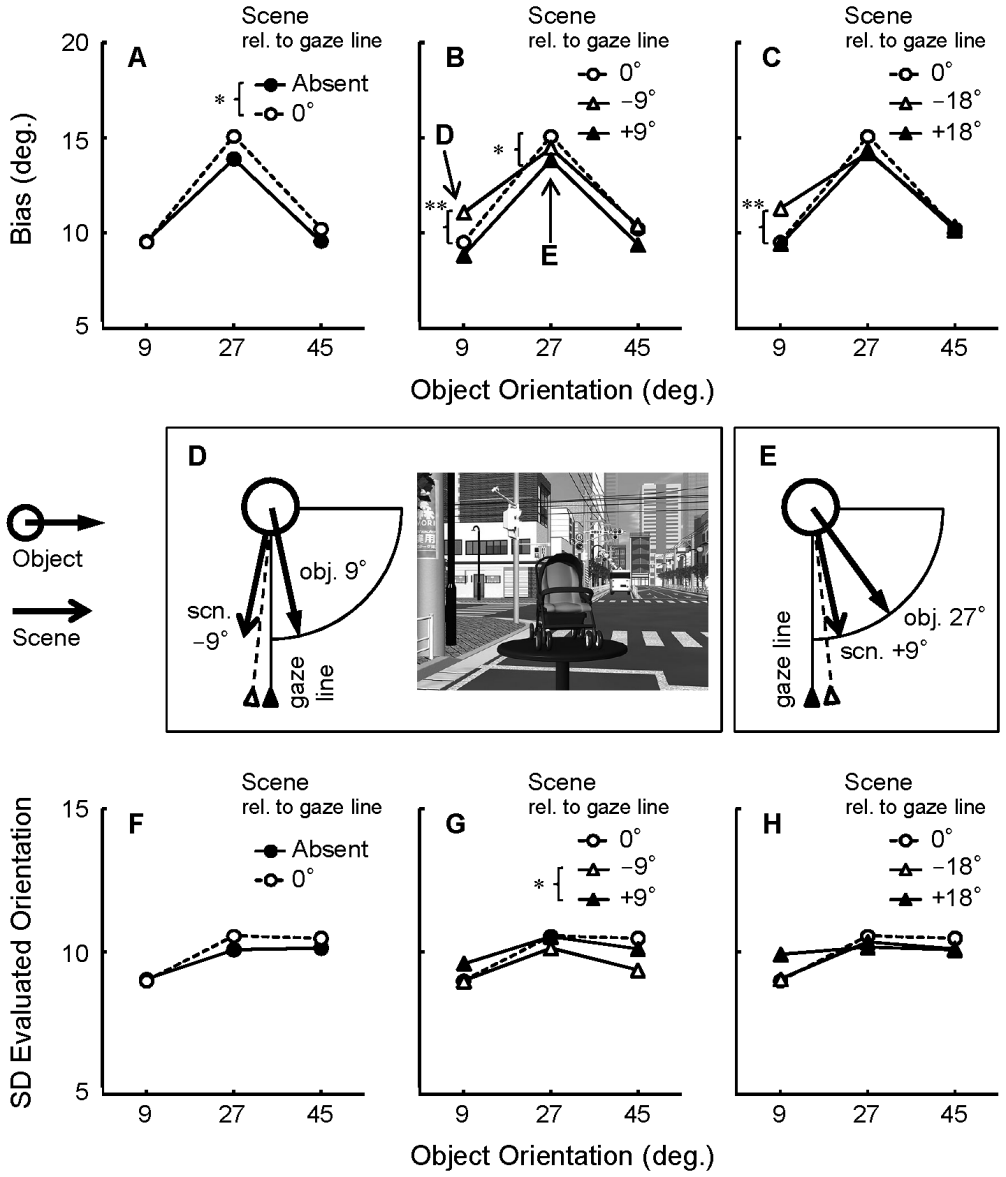
Results of the object orientation evaluation task in Experiment 1. Scene orientation was manipulated relative to the gaze line. Bias (evaluated orientation minus true object orientation) is plotted as a function of object orientation (A–C). The results of the Scene 0° condition are plotted repeatedly for comparison. Overall, a positive bias was observed, confirming the bias toward profile in the perception of oblique object orientations. Importantly, the bias was modulated by scene condition. Panels D and E illustrate top views of the spatial layouts of objects and scenes in the conditions indicated by the arrows in panel B. Dotted lines with open triangles indicate subjective gaze lines that might be biased toward the scene orientations. Panels F–G indicate the SD of the evaluated orientation as a function of object orientation. The ANOVA (object orientation × scene condition) was conducted for each of the panels A–C and F–G. The significant main effect and simple main effect of scene condition are marked by asterisks. **, *p*<.01, *, *p*<.05. Scn. = scene; obj. = object.

Figures 4A–4C show the mean bias as a function of object orientation. Consistent with the previous study [6], the mean bias was positive (i.e., toward profile) in all conditions. This bias was maximal for object orientations of 27°; on average, objects presented at orientations of 27° were perceived as if they were oriented at 41.3° (14.3° bias).

Our interest was confined to the effect of scene condition on bias in perceived object orientation. First, we compared the Scene 0° condition with the Scene Absent condition. As shown in Figure 4A, presenting a scene at 0° increased the bias slightly. Supporting this observation, a two-way repeated measures analysis of variance (ANOVA), with object orientation (9°, 27°, 45°) and scene condition (Absent, 0°) as factors, revealed a significant main effect of scene condition (*F*(1,18)  = 5.05, *p*<.05). The main effect of object orientation was significant as well (*F*(2,36)  = 8.26, *p*<.01), and post-hoc multiple comparisons using the Tukey's HSD confirmed that the objects oriented at 27° yielded the largest bias (*p*<.01). The interaction was not significant.

How consistent were the orientation evaluations? We analyzed the standard deviation (SD) of the evaluated orientation in the same way. For each participant, the SD of evaluated orientation for each condition was calculated. The SD values were averaged over participants and shown in Figure 4F. The two-way repeated-measures ANOVA did not report any significant effect (*p*>.05). Thus, object orientations and scene conditions influenced the mean evaluated orientation, but the SD did not.

Second, we tested the effect of misaligned scenes. The +/−9° conditions were compared with the 0° condition, which served as a baseline (Figure 4B). A two-way repeated measures ANOVA, with object orientation (9°, 27°, 45°) and scene condition (0°, +/−9°) as factors revealed significant main effects of scene condition (*F*(2,36)  = 14.94, *p*<.01) and object orientation (*F*(2,36)  = 8.52, *p*<.01). The interaction between object orientation and scene condition was also significant (*F*(4,72) = 2.70, *p*<.05). The simple main effect of scene condition was significant for object orientations of 9° and 27° (*p*<.01 and *p*<.05, respectively) but not 45° (*p* = .08). As indicated in Figure 4B, multiple comparisons of the simple main effects showed that the Scene −9° condition yielded a larger bias than the other scene conditions when the object orientation was 9° (*p*<.01), and that the Scene +9° condition yielded a smaller bias than the Scene 0° condition when the object orientation was 27° (*p*<.05).

The SD of the evaluated orientation is shown in Figure 4G. We found a significant main effect of scene condition (*F*(2,36) = 4.32, *p*<.05), while the main effect of object orientation and the interaction were not significant (*p*>.05). Multiple comparisons by the Tukey's HSD revealed that the Scene +9° condition yielded a larger SD than the Scene −9° condition (*p*<.05).

A comparable effect of scene condition on bias was found for scenes rotated +/−18°, in comparison to 0° scenes (Figure 4C). We observed a significant main effect of object orientation (*F*(2,36) = 7.73, *p*<.01) and a significant interaction (*F*(4,72) = 4.42, *p*<.01). The main effect of scene condition was not significant (*F*(2,36) = 2.14, *p* = .13). A simple main effect of scene condition was significant only for object orientations of 9° (*p*<.01), and multiple comparisons confirmed that scenes rotated −18° yielded a larger bias than the other scene conditions for object orientations of 9° (*p*<.01).

The same two-way ANOVA was conducted for the SD (Figure 4H), but no significant effect was found (*p*>.05).

### Discussion

The results of Experiment 1 suggest that global reference frames provided by visual scenes affect perceived object orientation, even though the scenes are task-irrelevant. First, the 0° scene reduced neither the bias nor the SD of the orientation evaluations (Figures 4A, 4F). This contradicts the hypothesis that the presence of a global reference frame would improve the accuracy of object orientation estimates. Therefore, the origin of the bias toward the profile, when estimating oblique orientations, is unlikely to be due to the lack of spatial information when stimuli are displayed on a blank background.

Second, visual scenes that were misaligned with the gaze line often modulated the perception of object orientation. When scenes were oriented at −9° or −18°, the bias in the orientation perception of objects at 9° increased (Figures 4B, 4C). One possible explanation for this result is that the misaligned global reference frames biased the egocentric reference frames (i.e., subjective orientation of the gaze line). Since the misalignment was not large (9° or 18°), the global and egocentric reference frames might have been confused. As schematized in Figure 4D, if the egocentric reference frame was assimilated into the scene axis, then the perceived object orientation relative to the egocentric reference frame would be overestimated, and thus bias would increase. This may also account for the reduced bias in the Object 27° condition, in the Scene +9° condition (Figure 4E). If the egocentric reference frame was assimilated into the +9° scene axis, the perceived object orientation relative to the egocentric reference frame would be biased, though the magnitude of the bias would decrease.

However, it is noteworthy that such assimilation effects were not always found in possible conditions; for example, there was no effect of scene orientation for objects orientated at 45°. The scene effects occurred only when the orientation of the object axis was similar to that of the scene. To further explore this issue, in Experiment 2, we examined the effect of global reference frames that were slightly misaligned with the object axes.

The analysis of SD (Figures 4G, 4H) also suggested that the misaligned scenes influenced the consistency of object orientation evaluation, although the influence did not appear to be systematic or robust.

## Experiment 2

The results of Experiment 1 suggest that scene orientation may bias the egocentric reference frame when their axes are close to each other, but slightly misaligned. In Experiment 2, we tested whether scenes that were slightly misaligned with the object axis would also affect the perception of object orientation. We manipulated the scene orientation relative to an object by −18°, −9°, 0°, +9°, or +18°. Otherwise, Experiment 2 was identical to Experiment 1. If a global reference frame that is slightly misaligned with the object axis influences the perception of that object's orientation, independent of the effect found in Experiment 1, then the magnitude of the bias will vary between scene conditions even when the scene and object orientations are not close to the gaze line orientation (e.g., 45° object orientation).

### Method

#### Participants

Eighteen individuals (mean age 21.8 years; range 18–45 years; 10 female, 8 male) were paid for their participation. They all reported normal or corrected-to-normal visual acuity, and had not participated in any other experiment reported in this paper.

#### Stimuli and Apparatus

The object, scenes, and apparatus were the same as those used in Experiment 1.

#### Design and Procedure

The task was the same as in Experiment 1. Object orientations were also identical to those used in Experiment 1 (9°, 27°, and 45°; left and right), though instead we manipulated the scene orientation relative to the object orientation (0°, +/−9°, and +/−18°). Note that the scene orientations here indicate orientation differences between the scene and the object, not differences between the scene and the gaze line. Hence, the Scene 0° condition indicated that the scene axis was aligned with the object axis. For example, when the object orientation was 9° right (left), Scenes −18°, −9°, 0°, +9°, and +18° conditions indicated that the scene was oriented 9° left (right), 0°, 9° right (left), 18° right (left), and 27° right (left), respectively. If scenes that are misaligned with the object affect the perception of the object's orientation, the magnitude of the bias will vary between scene conditions. The Scene Absent condition was also included, as in Experiment 1.

Each participant performed 648 trials. Trial order was randomized. Prior to the experimental trials, participants completed 12 practice trials. The experiment lasted approximately 80 minutes.

### Results

Bias was analyzed in the same manner as in Experiment 1; the left/right difference was ignored; if an absolute value of bias was larger than 45°, then the trial was regarded as an error. We omitted data from two participants who had exceptionally high error rates (>10%). The mean error rate for the remaining 16 participants was 0.34%. Error trials were excluded from the following analyses.

**Figure 5:**
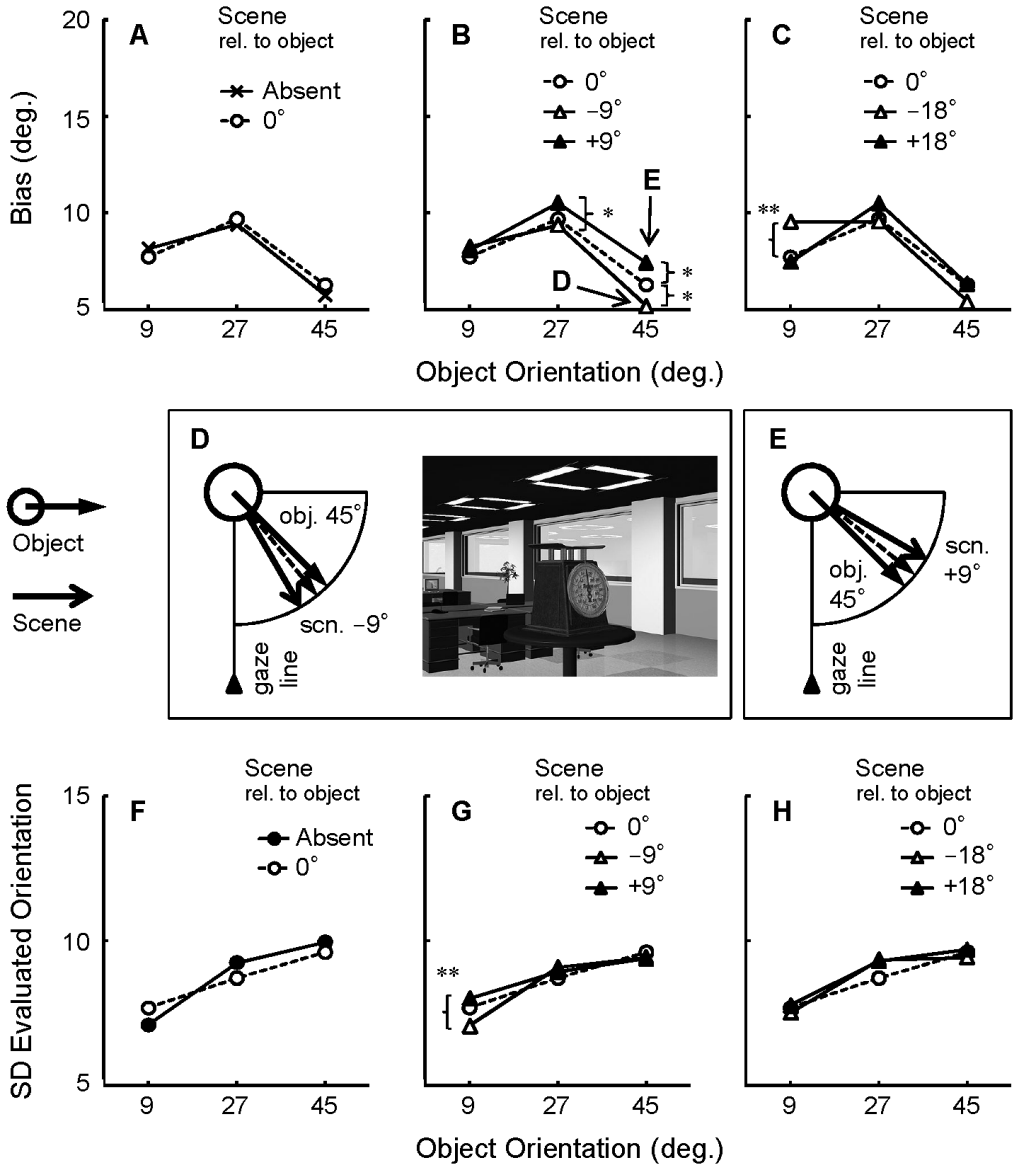
Results of the object orientation evaluation task in Experiment 2. Scene orientation was manipulated relative to the object. The bias (evaluated orientation minus object orientation) is plotted as a function of object orientation (A–C). The results from the Scene 0° condition are plotted repeatedly for comparison. The bias toward profile was confirmed just as in Experiment 1. Further, +/−9° scenes modulated the magnitude of this bias (B). The spatial layouts of the conditions in which +/−9° scenes affected the results are schematized in panels D and E. As indicated by the dotted arrows, object orientations were evaluated as if they were assimilated into the scene orientations. Panels F–G indicate the SD of the evaluated orientation as a function of object orientation. The ANOVA (object orientation × scene condition) was conducted for each of the panels A–C and F–G. The significant main effect and simple main effect of scene condition are marked by asterisks. **, *p*<.01, *, *p*<.05. Scn. = scene; obj. = object.

First, we contrasted the Scene 0° condition with the Scene Absent condition to assess the effect of an object-aligned scene. As shown in Figure 5A, the 0° scene had no effect. The two-way repeated-measures ANOVA (object orientation × scene condition) found a significant main effect of object orientation (*F*(2,30) = 6.35, *p*<.01); there was no significant main effect of scene condition (*F*<1) or a significant interaction (*F*(2,30) = 1.93, *p* = .16). Although object-aligned scenes might provide rich allocentric spatial information, they did not reduce the bias in estimating oblique object orientations.

The SD of the evaluated orientation for the Scene 0° and Scene Absent conditions is shown in Figure 5F. The main effect of object orientation (*F*(2,30) = 16.18, *p*<.01) and the interaction effect (*F*(2,90) = 3.44, *p*<.05) were significant. This might imply that there exist slightly different patterns of object orientation as a function of scene presence. However, the simple main effect of scene condition was not significant (*p*>.05) for any of the object orientations.

Second, we contrasted the misaligned scene conditions with the Scene 0° condition. When the misalignment between scene and object was small (+/−9°), the perceptual bias was influenced by the scene in the Object 27° and 45° conditions (Figure 5B). This was confirmed by the two-way repeated-measures ANOVA (object orientation × scene condition) that revealed significant main effects of object orientation (*F*(2,30) = 6.17, *p*<.01), scene condition (*F*(2,30) = 10.00, *p*<.01) and interaction (*F*(4,60) = 3.76, *p*<.01). Multiple comparisons of the simple main effects of scene condition revealed the significant differences marked by the asterisks in Figure 5B. In sum, the −9° scenes reduced perceptual bias, while the +9° scenes increased it.

Figure 5G shows the SD for the Scene +/−9° conditions. The ANOVA revealed that the main effect of object orientation (*F*(2,30) = 13.38, *p*<.01) and the interaction effect (*F*(4,60) = 3.11, *p*<.05) were significant. The simple main effect of scene condition was significant for a 9° object orientation (*p*<.01) but not for other object orientations.

However, the effect of scene on the bias was not found when the scene misalignment was large (+/−18°, see Figure 5C). The two-way repeated measures ANOVA revealed a significant main effect of object orientation (*F*(2,30) = 7.19, *p*<.01) and a significant interaction (*F*(4,60) = 5.70, *p*<.01), but the main effect of the scene condition was not significant (*F*<1). The interaction reflects the significantly larger bias in the Scene −18° condition for the Object 9° condition (*p*<.01). This effect replicates Experiment 1; in this condition, the object orientation was 9° left (right) and the scene orientation was 9° right (left). The spatial layouts coincided with the Object 9° and Scene −9° condition in Experiment 1 (Figure 4D).

The ANOVA on the SD of the evaluated orientation (Figure 5H) revealed that the main effect of object orientation was significant (*F*(2,30) = 11.78, *p*<.01). Neither the main effect of scene condition nor the interaction effect was significant.

### Discussion

The results of Experiment 2 show that scenes that are slightly (9°) misaligned with an object axis will bias the perception of that object's orientation. Global reference frames may influence the perception of object orientation when they are slightly misaligned with either gaze line (Experiment 1) or object axis (Experiment 2).

Object orientations were perceived as if they were assimilated into the scene orientations, as schematized in Figures 5D and 5E. This effect was qualitatively different from that found in Experiment 1; if the same phenomenon had occurred in Experiment 1, the bias in the condition shown in Figure 4D would be reduced because the perceived object orientation would be assimilated into the scene orientation.

The assimilation effect found here implies that participants confuse object and scene orientation. Supporting this view, the larger (18°) misalignments between the scene and object did not produce such perceptual modulation; perhaps a misalignment of 18° was sufficient to permit discrimination between the object and scene axes, whereas a misalignment of 9° was not. However, the assimilation effect when the scene was misaligned by 9° was absent when the object orientation was near the front (9°). These effects are examined further in Experiment 3.

The SD of the evaluated orientation was significantly modulated by object orientation, irrespective of scene condition. As seen in Figures 5F–5H, the object orientations closer to the front yielded a smaller SD. The result seemed confusing because the SD of the evaluated orientation was rather independent of object orientation in Experiment 1 (Figures 4F–4H). The scene conditions did not influence the SD very reliably. The only effect we found was the reduced SD in the Scene −9° condition for the evaluations of the 9° object orientation (Figure 5G), which seemed trivial.

## Experiment 3

Our previous study [2] showed that it is easier to discriminate between an oblique orientation and a frontal orientation (e.g., 15° vs. 0°) than between two oblique orientations (e.g., 60° vs. 45°). Therefore, it was plausible that, in Experiment 2, scene misalignments of +/−9° in the Object 9° condition were subjectively apparent, while scene misalignments of +/−9° in the Object 45° condition were not. If this is the case, the lack of assimilation effect in the Object 9° condition (Figure 5B) might reflect the fact that orientation differences between object and scene were easily discriminated. To test this hypothesis, we measured the discriminability between object and scene orientation, and tested whether it depended on object orientation.

### Method

#### Participants

Sixteen individuals (mean age 22.1 years; range 18–27 years; 11 male, 5 female) were paid for their participation. All reported normal or corrected-to-normal visual acuity, and had not participated in any other experiment reported in this paper.

#### Stimuli and Apparatus

The stimuli (objects, scenes) and apparatus were the same as in the previous experiments, except that the response display was replaced with a keypad.

#### Design and Procedure

The task was to judge whether the object axis deviated leftward or rightward from the scene axis. Participants reported their judgments by pressing the appropriate key in a dual alternative (left or right) forced-choice manner. For example, when the object was oriented 27° right and the scene was oriented 36° right, relative to the gaze line, then the correct response was “right”; however, if the scene was oriented 18° right, relative to the gaze line, then the correct response was “left.” We instructed participants to respond as accurately as possible.

The conditions were identical to Experiment 2, except that the Scene 0° and Scene Absent conditions were not included. Hence, we examined the effects of object orientation (9°, 27°, 45°; left/right) and scene orientation, relative to the object (−18°, −9°, +9°, +18°), on discrimination performance. All conditions were randomized.

### Results and Discussion

We omitted data from two participants whose average accuracy was below chance (.5). The results for the remaining 14 participants are reported below.

**Figure 6:**
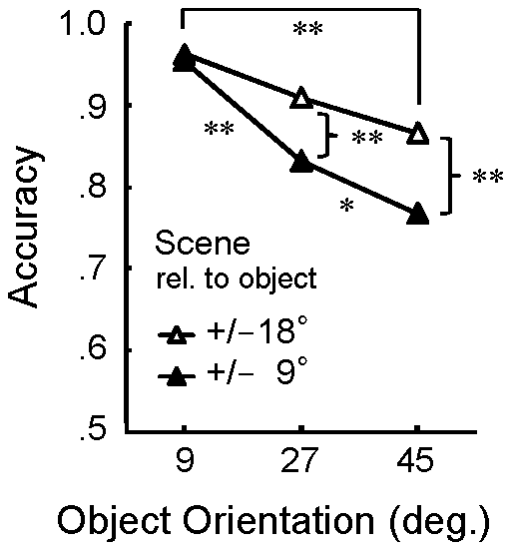
Results of Experiment 3. Participants were asked to report the direction of misalignment between the object and scene axes. Scene orientation was misaligned +/−9° or +/−18° with respect to object orientation. Scene-object misalignments were more easily perceived for objects oriented at 9° than for objects oriented at 45°.

The mean accuracy was calculated for each condition (see Figure 6). In the same manner as Experiments 1 and 2, we merged the symmetric conditions. We also ignored the direction of scene orientation misalignment (positive or negative). A two-way repeated measures ANOVA was conducted, with object orientation (9°, 27°, 45°) and scene condition (+/−9°, +/−18°) as factors. The main effect of scene condition was significant (*F*(1,13) = 49.15, *p*<.01), such that accuracy was higher when misalignment was greater. The main effect of object orientation and the interaction were also significant (*F*(2,26) = 24.58, *p*<.01; *F*(2,26) = 9.21, *p*<.01, respectively). The interaction is likely due to a ceiling effect in the Object 9° condition. Multiple comparisons of the simple main effects of object orientation confirmed that, in both scene conditions, accuracy was higher in the Object 9° condition than the Object 45° condition (*p*<.01).

The misalignment of the scene axis relative to the object axis was detected more easily for the near-front object orientation (9°) than for the oblique object orientation (45°), even when the degree of misalignment was constant. This supports the hypothesis that the differential effect of scene misalignment on the perception of object orientation (Experiment 2, see Figure 5B) may be related to a lower orientation discriminability between the object and scene axes.

## General Discussion

We found that (a) background scenes did not improve object orientation perception compared to a blank background; and (b) scene orientation influenced object orientation perception when the scene axis was close to (but misaligned with) either gaze line or object axis. These findings suggest that a global reference frame has a small but reliable influence on the object orientation judgments based on an egocentric reference frame in 3D space.

Previously we reported that the perception of oblique object orientation is biased toward the profile [6] and relatively inefficient [2]. Experiment 1 showed that the presence of gaze-aligned scene reduced neither bias (Figure 4A) nor variability in evaluated orientation (Figure 4F). The biased perception of oblique object orientations for objects presented on a blank background was not likely due to a lack of global spatial information.

We found several cases where the scene biased the perceived object orientation (Figure 4B, 4C, 5B, and 5C), even though the task was to evaluate object orientation based on an egocentric reference frame, and the scene was task-irrelevant. These findings indicate that global reference frames exert a contextual effect on the perception of orientation in 3D space. Thus the three reference frames—egocentric, global, and object-centered—are not processed separately, but interact with each other.

Experiments 2 and 3 revealed that the contextual effect of scene emerged when the scene axis was slightly misaligned with either gaze line or object axis. The gaze line and object axis orientations may have been assimilated into the adjacent scene orientation. As suggested by Experiment 3, this effect occurs when the misalignment is less salient, or in other words, when the global reference frame is easily confused with the other reference frames. The visual system may be inclined to ignore small misalignments between reference frames, and to perceive that the objects and gaze line are aligned with the global reference frame.

The assimilation effect observed here may be related to uncertainty regarding object orientation. A study on 3D shape perception reported that the perceived shape of a 3D object, presented in noise, was assimilated into the shapes of surrounding objects [30]. Similarly, ambiguity in shape orientation [14] or object-motion direction [31] can be resolved using contextual information. The perception of oblique object orientation is inaccurate and uncertain. Thus, it is likely that the visual system utilizes scene orientation as a contextual cue to guesstimate an object's orientation, as long as the object is not obviously misaligned with the scene axis.

How does the ‘assimilation’ of reference frames occur? In the Scene Absent condition, the object orientation evaluation is based solely on the object image, which contains visual information such as perspective cues. The assimilation of object orientation to the scene in Experiment 2 (Figures 5D, E) is likely to occur because visual attention to the object does not completely filter out the visual information of the scene (e.g., perspective cue of the room). However, this account does not fit the assimilation of egocentric reference frame to the scene in Experiment 1 (Figures 4D, E). If the visual system simply confused the perspective cues of object and scene, the object orientation evaluation should be assimilated toward scene orientation, but the actual effect was in the opposite direction. Hence, the effect may be attributable to higher-order mechanisms: the egocentric reference frame was biased by the (misaligned) scene orientation, or the visual system confounded object orientation relative to the egocentric reference frame and object orientation relative to scene. Although the effects of the background scene were understandable, they were not prominent. For instance, in Experiment 1, the bias increment between the -9° scene and the 0° scene (see Figure 4B, 4D) was 1.54°, which is small relative to the size of the bias (10∼15°). Furthermore, the effects of scene were found only under limited conditions. The role of the scene context in 3D object orientation perception may be supplemental rather than essential. Nevertheless, the presence of a scene effect found here is a hallmark of scene processing in object orientation perception. The scene effects may likely be more prominent in more uncertain conditions, for example, when evaluating the orientation of a bar-shaped object, which provides less pictorial depth information than the more familiar objects we used.

As a result of using “flat” 2D stimuli, the background scene effect we found was attributable to pictorial depth information. Other types of depth information that “real” 3D stimuli provide, such as binocular disparity, might modulate the effect. If the effect we found was purely pictorial (e.g., the assimilation effect would only occur when the oblique edges adjacent to the object were present), the binocular disparity of 3D stimuli may reduce the effect. In contrast, if the effect of the background originated from the perceived global reference frame, the binocular disparity may emphasize the influence of the global reference frame and actually increase the effect. This issue should be examined further.

In addition, the presence of the CRT display frame surrounding the stimulus images (see Figure 2A) might provide a cue of the flatness of the stimuli. It was reported that the presence of a frame increased the bias of the perceived slant orientation even when the stimuli were “real” 3D objects [7], [32]. Therefore, it is possible that the magnitude of bias we observed was partly exaggerated by the CRT frame. However, it is unlikely that the flatness cue produced the effects of background scenes, such as the assimilation effect, although it might have increased the bias overall.

In conclusion, we confirmed that visual scenes exert contextual effects on the perception of object depth orientation in 3D space. This effect is not large in size, but analogous to the contextual effect of a tilted background scene on 2D orientation perception [17]–[19]. Although a global reference frame is inexorably processed when observers try to determine object orientation, it might not facilitate the valid perception of object orientation. Rather, its functional role may be more related to contextual modulation of object orientation perception under relatively uncertain conditions.

## Supporting Information

Figure S1 **The 18 stimulus objects for the experimental trials, shown in the object orientation of 45° left.** The top row shows wide objects; middle, high objects; bottom, deep objects.
(JPG)
