# Supplementary figures and images for: Contextual Effects of Scene on the Visual Perception of Object Orientation in Depth
Source: PLoS One. 2013 Dec 31;8(12):e84371. doi: 10.1371/journal.pone.0084371 (PMC3877279; doi:10.1371/journal.pone.0084371)

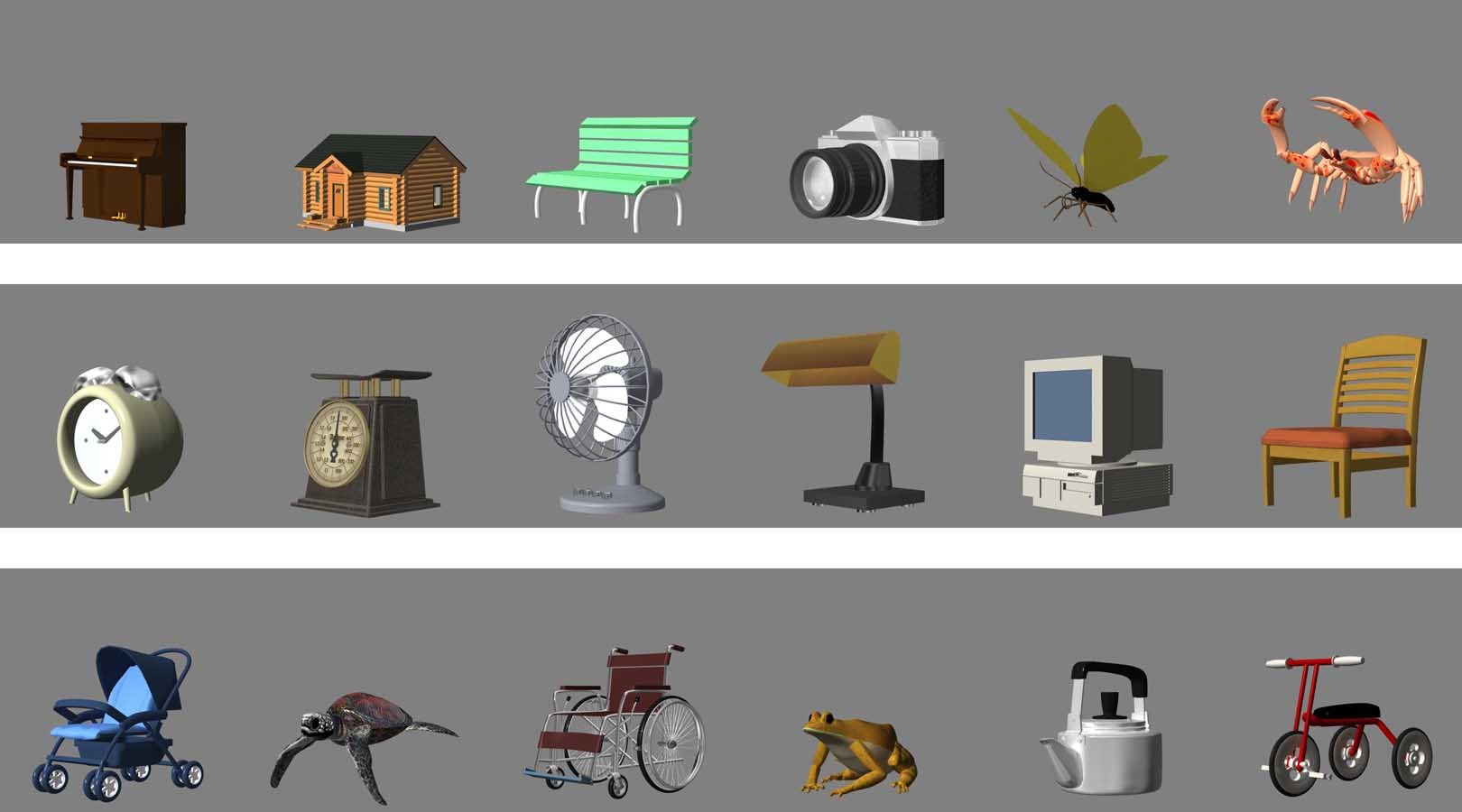

Supplement: Figure S1 — The 18 stimulus objects for the experimental trials, shown in the object orientation of 45° left. The top row shows wide objects; middle, high objects; bottom, deep objects. (JPG) [file pone.0084371.s001.jpg]
